# Efficacy and Safety of Analgesics and Sedatives during Radiofrequency Catheter Ablation of Atrial Fibrillation: A Network Meta-Analysis

**DOI:** 10.31083/j.rcm2501012

**Published:** 2024-01-09

**Authors:** Le Jin, Fang Liu, Hongmei Gao, Luyao Zheng

**Affiliations:** ^1^The Second Clinical College, Shandong University of Traditional Chinese Medicine, 250014 Jinan, Shandong, China; ^2^Department of Cardiology, The Second Affiliated Hospital of Shandong University of Traditional Chinese Medicine, 250001 Jinan, Shandong, China

**Keywords:** atrial fibrillation, radiofrequency catheter ablation, analgesia, sedation

## Abstract

**Background:**

Atrial fibrillation is the most common tachyarrhythmia, while catheter ablation is an effective therapy for atrial fibrillation. However, pain and nervousness may occur during the procedure. Moreover, a consensus has still not been reached on which is the best kind of analgesic and sedative to use in these procedures. Therefore, we conducted a network meta-analysis to evaluate the efficacy and safety of analgesics and sedatives used in catheter ablation for atrial fibrillation.

**Methods:**

We searched PubMed, Cochrane Library, Web of Science, EMBASE, China National Knowledge Infrastructure, and Baidu Wenku document download website for randomized controlled trials from their inception to February 26, 2023. Only studies that made comparisons among analgesics or sedatives and involved patients with atrial fibrillation undergoing radiofrequency catheter ablation were included. The efficacy endpoints were Ramsay sedation scores and visual analog scale scores during the radiofrequency catheter ablation for atrial fibrillation. The safety endpoints were the incidence of respiratory depression, hypotension, nausea, and vomiting. Pairwise comparisons and frequency method analyses were conducted. Results were reported as odds ratio (OR), mean difference (MD), and corresponding 95% confidence intervals (CIs). We assessed the risk bias of the studies in accordance with the Cochrane Handbook for Systematic Reviews of Interventions.

**Results:**

Out of the 709 articles initially retrieved, 14 studies, with a total of 1156 participants, were included. In terms of efficacy, patients receiving dexmedetomidine during radiofrequency ablation for atrial fibrillation had higher Ramsay sedation scores than those receiving midazolam plus fentanyl, or its derivatives (MD –0.88, 95% CI [–0.04 to –0.72]). Compared with morphine, dezocine (MD 1.88, 95% CI [1.16 to 2.60]), hydromorphone (MD 4.07, 95% CI [3.56 to 4.58]), butorphanol (MD 3.18, 95% CI [2.38 to 3.96]), and fentanyl or its derivatives (MD 1.57, 95% CI [1.25 to 1.89]) had a better analgesic effect. In terms of safety, propofol (OR 16.46; 95% CI [1.54 to 175.95]) and midazolam plus fentanyl or its derivatives (OR 7.02; 95% CI [1.33 to 36.99]) significantly increased the incidence of respiratory depression compared with dexmedetomidine plus fentanyl or its derivatives. Dexmedetomidine plus fentanyl or its derivatives reduced the incidence of nausea and vomiting compared with fentanyl alone (OR 4.74; 95% CI [1.01 to 22.22]). Propofol was associated with a lower incidence of nausea and vomiting than hydromorphone (OR 0.01; 95% CI [0.00 to 0.59]) and fentanyl or its derivatives (OR 0.01; 95% CI [0.00 to 0.51]). There was no statistically significant difference in the incidence of hypotension between any two strategies.

**Conclusions:**

Hydromorphone and butorphanol had better analgesic effects than fentanyl or its derivates. Dexmedetomidine had better sedative effects. In terms of safety, dexmedetomidine, oxymorphone, and butorphanol were superior. It is necessary to explore the regimen that can consider both the effectiveness and safety during radiofrequency catheter ablation for atrial fibrillation (AF).

**The PROSPERO Registration:**

This study was registered with PROSPERO, number: CRD42023403661.

## 1. Introduction 

Atrial fibrillation (AF) is a common tachyarrhythmia, which increases in incidence each year. Radiofrequency catheter ablation (RFA) is a minimally invasive procedure for the treatment of AF. At present, RFA in patients with AF has become an important treatment method. Randomized controlled trials (RCTs) have shown that RFA for AF has advantages over drug therapy in the treatment of paroxysmal and persistent atrial fibrillation [1]. A catheter is placed into the atrium through venipuncture, and three-dimensional modeling of the atrium is carried out, with the assistance of the CARTO three-dimensional mapping system (Biosense Webster, Inc., Diamond Bar, CA, USA), to search for the substrate causing AF and apply high-frequency current for the ablation of myocardial tissue, to ablate the arrhythmia. However, high-frequency currents will cause some thermal damage to the myocardium during the procedure, and the three-position mapping system requires patients to maintain a certain position for a long time. As a result, patients may be unable to tolerate the pain or move their limbs. This can lead to the displacement of three-dimensional images, thus, affecting the accuracy of the ablation targets, prolonging the operation time, and increasing the possibility of postoperative complications. Therefore, the implementation of a safe and effective sedation and analgesia strategy is crucial to ensure the success of RFA procedures.

The main sedative and analgesic drugs used in RFA for patients with atrial fibrillation include opioids, benzodiazepines, α_2_ adrenergic receptor agonists, and propofol. Most sedative and analgesic drugs have the potential for adverse reactions. At present, there are several high-quality RCTs, which have compared sedative or analgesic strategies. However, there are still various studies that are focusing on which sedative and analgesic strategies can combine safety and efficacy in RFA for atrial fibrillation. Therefore, we conducted a network meta-analysis based on the frequency framework to compare the effectiveness and safety of these different sedation or analgesia regimens in RFA for AF.

## 2. Methods

We followed the Preferred Reporting Items for Systematic Reviews and Meta-Analyses (PRISMA) guidelines. Additionally, we developed a protocol and registered it on PROSPERO (CRD42023403661).

### 2.1 Search Strategy and Selection Criteria

We performed a systematic review and network meta-analysis. We searched the PubMed, Cochrane Library, Web of Science, EMBASE, China National Knowledge Infrastructure, and Baidu Wenku document download website for randomized controlled trials from the date of their inception to Feb 26, 2023, without language restrictions. To ensure the comprehensiveness of the retrieval, a combination of subject terms and free terms was used for the literature retrieval. All search results were stored using Endnote software (Thomson Corporation, Stanford, CT, USA) for further filtration. We used the following search keywords: “atrial fibrillation”, “auricular fibrillation”, “AF”, “analgesia”, “analgesic”, “sedation”, “sedative”, “radiofrequency ablation”, and “catheter ablation”, which were combined with analgesic and sedative drugs currently used in clinical practice, such as “morphine” and “fentanyl”. We only included RCTs that compared different analgesic or sedative strategies in patients with AF undergoing RFA.

The inclusion criteria were as follows: (1) patients diagnosed with AF undergoing RFA; (2) study design was an RCT to compare different analgesics or sedatives during RFA; (3) study with outcomes of “Ramsay sedation scores (RSS)”, “visual analog scale (VAS)”, “incidence of respiratory depression”, “incidence of hypotension”, or “incidence of nausea and vomiting”; (4) studies with two or multiple arms.

The exclusion criteria were as follows: (1) study types were reviews, observational studies, registry data, ongoing trials without results, case reports, systematic reviews, meta-analysis, animal experimental studies, or duplicate studies; (2) studies without definition of endpoints; (3) unrelated studies; (4) study data could not be obtained.

### 2.2 Outcome

Our endpoints were the efficacy of an analgesic or sedative, evaluated using RSS and VAS scores. The RSS was defined: one point: the patient is awake but anxious, agitated, or restless; two points: the patient is awake but cooperative, orientated, and tranquil; three points: the patient is drowsy but responsive to commands; four points: the patient is asleep and with brisk response to glabella tap or loud auditory stimulus; five points: the patient is asleep with sluggish response to a stimulus; six points: the patient has no response to noxious stimuli. Pain was assessed by VAS, using a scale of 0 to 10. The VAS rules were as follows: higher scores represent more intense pain, a score of 0 represents no pain, and 10 points represent severe pain. The safety endpoints were the incidences of respiratory depression, hypotension, nausea, and vomiting during ablation therapy. The trial defined respiratory depression as ${\mathrm{SaO}}_{2}$ <90%, apnea greater than 15 seconds, respiratory rate less than 8 breaths per minute, or hypercarbia (${\mathrm{PaCO}}_{2}$ >55 mmHg). Hypotension was defined as a systolic pressure of less than 90 mmHg or mean arterial pressure of less than 60 mmHg.

### 2.3 Data Extraction and Quality Assessment

The search results were screened independently by two blinded researchers (LJ and LZ), according to inclusion and exclusion criteria. Disagreements were resolved through discussions and referral to a third coauthor (HG). Two authors independently extracted the following data from the included RCTs: the first author’s name, the publication year, the baseline characteristics, the number of patients in each group, interventions, procedure duration, Ramsay sedation scores, analgesic scores, and occurrence of adverse reactions.

Two authors independently accessed the quality and risk of bias of the included studies using the Cochrane Collaboration Systematic Evaluators manual. We used Review Manager software (Version 5.4, Cochrane, London, United Kingdom) to produce the risk of bias graph. To assess publication bias, we generated funnel graphs using STATA software (Version 17.0, StataCorp, Texas City, TX, USA).

### 2.4 Statistical Analysis

The data were analyzed using STATA software (Version 17.0) based on a frequency model. First, we drew network evidence plots to show direct comparisons. We plotted network contributions to show the contribution of direct comparisons to indirect comparisons. We used “ifplot” in the “network” software package (Thomson Corporation, Stanford, CT, USA) to evaluate inconsistencies. The inconsistency factor (IF) and IF 95% confidence interval (CI) were used to evaluate differences between direct and indirect comparisons. If 95% CI of IF contains 0, the consistency is high. According to the inclusion and exclusion criteria, only RCTs were included in the network meta-analysis, meaning there was no loop formed regarding the efficacy endpoints and the incidence of hypotension, nausea, and vomiting. An inconsistency test was not required to evaluate the results of the direct comparison and indirect comparison. Then, interval plots were drawn. We performed league tables to show the network meta-analysis (NMA) results. To compare each strategy, we used the surface under the cumulative ranking curve (SUCRA) to estimate the probabilities (%) of each treatment, as being the best or other rankings. Finally, we developed correction funnel plots to determine the evidence of the small sample effect and publication bias.

## 3. Results

### 3.1 Literature Selection

**Fig. 1.**
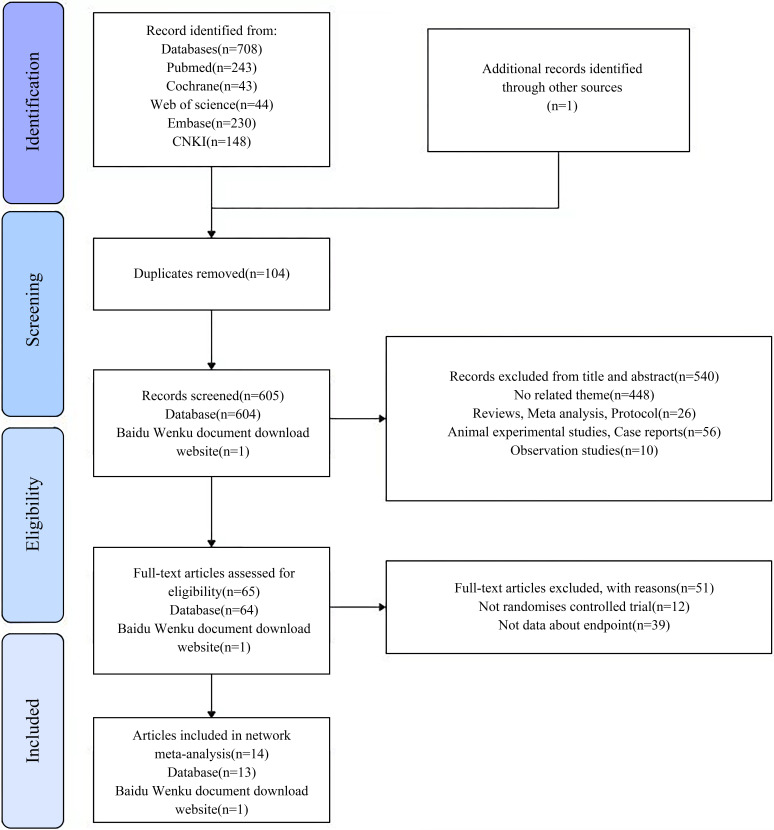
**The Preferred Reporting Items for Systematic Reviews and Meta-Analyses (PRISMA) flowchart for this network meta-analysis**. CNKI, Chinese National Knowledge Infrastructure.

**Fig. 2.**
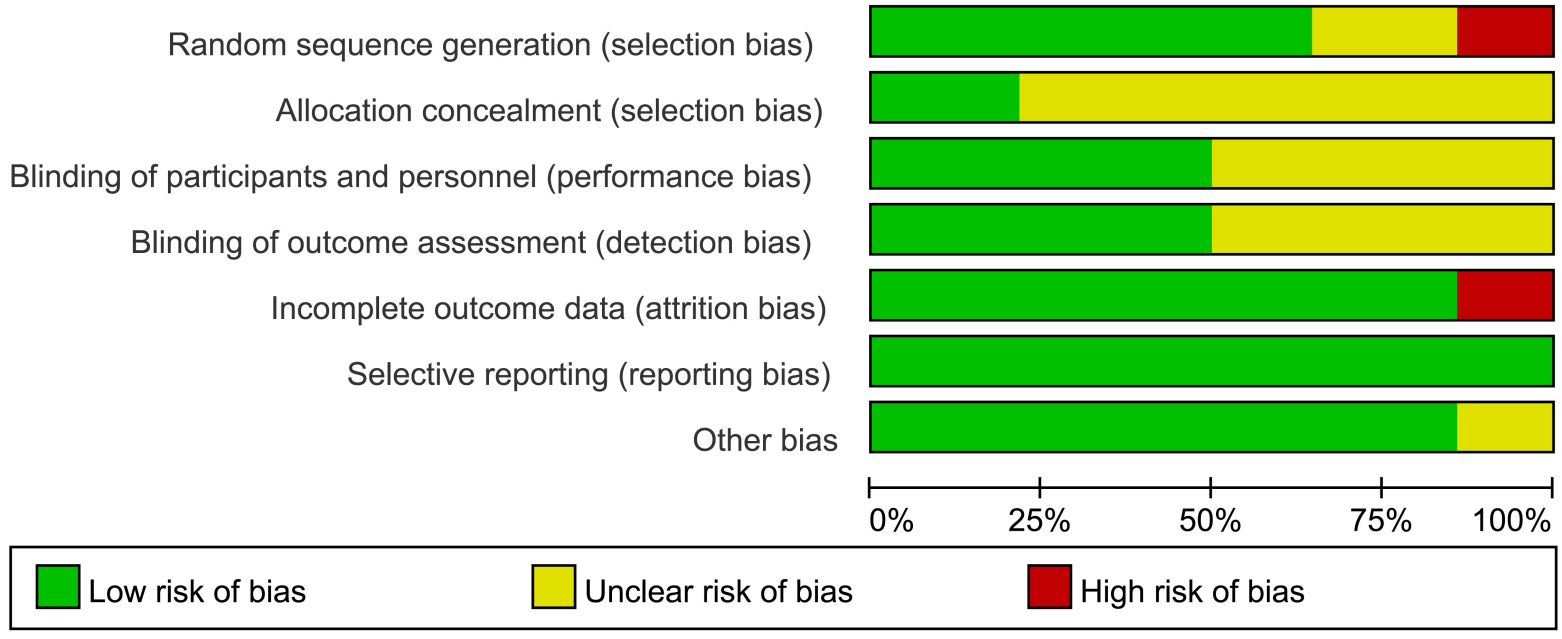
**Risk of bias graph**.

We retrieved 708 articles from the PubMed, Cochrane Library, Web of Science, EMBASE, and China National Knowledge Infrastructure databases, and 1 article from the Baidu Wenku document download website. A total of 104 duplicate articles were excluded. After reading the titles and abstracts, 540 articles were removed, and 51 articles were further screened by reading the full text. Finally, 14 RCTs were included in the NMA, including 13 from the database and 1 from the Baidu Wenku document download website. Fig. 1 illustrates the literature screening process. The 14 studies [2, 3, 4, 5, 6, 7, 8, 9, 10, 11, 12, 13, 14, 15] involved 1156 patients, 9 drugs, and 10 regimens, which were propofol, dexmedetomidine, fentanyl or its derivatives, butorphanol, oxycodone, hydromorphone, dezocine, morphine, dexmedetomidine combined with fentanyl or its derivatives, and midazolam combined with fentanyl or its derivatives. The studies and the patient characteristics are shown in Table 1 (Ref. [2, 3, 4, 5, 6, 7, 8, 9, 10, 11, 12, 13, 14, 15]). All included studies were RCTs. The risk of bias and quality evaluation results for the studies are shown in Fig. 2.

**Table 1.** **Basic characteristics of included studies**.

Study	Intervention		Sample size		Age		Gender (female/male)		Weight (kg)		ASA (I/II/III)		Procedure time (min)		Outcomes reported	Race	Study setting
	CG	EG	CG	EG	CG	EG	CG	EG	CG	EG	CG	EG	CG	EG			
Servatius H, *et al*. 2022 [2]	PRO	DEX	80	80	64.2 ± 11.9	65.5 ± 69.6	23/57	28/52	—	—	0/66/14	0/58/22	—	—	②	European	Single-center, single-blind
Cho JS, *et al*. 2014 [3]	DEX+F	MD+F	45	45	55.2 ± 8.7	56.3 ± 9.3	36/9	36/9	70.9 ± 9.6	72.9 ± 12.2	16/29/0	8/37/0	199.7 ± 36.7	210.1 ± 48.7	①②④	Asian	Sigle-center, single-blind
Tang RB, *et al*. 2007 [4]	PRO	MD+F	60	60	—	—	—	—	—	—	—	—	—	—	②④	Asian	Sigle-center, single-blind
Gu XK, 2020 [5]	F	BT	40	40	58.7 ± 3.6	57.4 ± 3.5	12/28	14/26	73.2 ± 5.9	70.0 ± 5.2	24/16/0	26/14/0	160.6 ± 19.8	159.8 ± 23.3	①②③④	Asian	Sigle-center, single-blind
Chang EQ, *et al*. 2020 [8]	F	OXY	50	50	54.8 ± 6.4	52.5 ± 8.9	27/21	29/23	66.0 ± 9.3	67.9 ± 4.1	19/32/0	18/31/0	—	—	②	Asian	Sigle-center, double-blind
Ding N, *et al*. 2018 [9]	F	HMOR	30	30	65.3 ± 6.7	65.4 ± 6.6	14/16	12/18	66.5 ± 9.0	69.7 ± 6.2	—	—	120.4 ± 30.4	126.2 ± 29.5	①②③④	Asian	Sigle-center, double-blind
Ni WJ, 2020 [10]	MD+F	DEX+F	23	25	61.6 ± 9.1	63.2 ± 9.1	13/10	17/8	70.8 ± 10.1	74.5 ± 8.6	—	—	151.3 ± 39.9	142.8 ± 33.9	①②④	Asian	Sigle-center
Yuan SP, *et al*. 2021 [11]	F	DEX+F	52	43	55.3 ± 13.8	54.3 ± 11.1	30/22	25/18	—	—	—	—	213.4 ± 23.6	217.2 ± 21.3	①②	Asian	Sigle-center
Long XF, *et al*. 2017 [12]	F	DEX+F	40	40	58.5 ± 4.7	59.2 ± 4.2	25/15	26/14	60.4 ± 8.6	62.6 ± 7.3	21/19/0	23/17/0	218.1 ± 20.3	212.4 ± 25.3	①②④	Asian	Sigle-center, triple blind
Yuan JF, *et al*. 2014 [13]	F	DEX+F	30	30	50-65	50-65	—	—	—	—	—	—	220.0 ± 7.1	206.0 ± 4.6	①④	Asian	Sigle-center
Chen HY, *et al*. 2018 [14]	DEX	MD+F	24	24	46.0 ± 7.0	48.0 ± 6.0	18/6	20/4	—	—	—	—	—	—	①②	Asian	Sigle-center
Li KY, *et al*. 2021 [15]	MOR	F	40	40	66.7 ± 7.4	65.7 ± 6.3	22/18	23/17	73.0 ± 11.7	70.7 ± 10.6	—	—	96.9 ± 19.3	104.0 ± 21.3	①②③④	Asian	Sigle-center
Tan J, *et al*. 2016 [6]	MOR	DZ	30	60	60.1 ± 15.1	59.3 ± 14.3	20/10	40/20	66.0 ± 30.1	65.1 ± 29.3	—	—	252.4 ± 41.2	202.3 ± 39.1	③	Asian	Sigle-center
Li FZ, *et al*. 2015 [7]	MOR	DZ	20	25	54.6 ± 12.5	61.0 ± 15.3	13/7	12/13	—	—	—	—	258.0 ± 77.5	194.4 ± 38.0	③	Asian	Sigle-center

CG, control group; EG, experimental group; PRO, propofol; DEX, dexmedetomidine; F, fentanyl or its derivatives; BT, butorphanol; OXY, oxycodone; HMOR, hydromorphone; MOR, morphine; MD, midazolam; DZ, dezocine; ASA, American Society of Aneshesiologists. ①: intraoperative sedation scores; ②: incidence of respiratory depression; ③: intraoperative analgesic scores; ④: incidence of nausea and vomiting.

### 3.2 Efficacy Endpoints

**Fig. 3.**
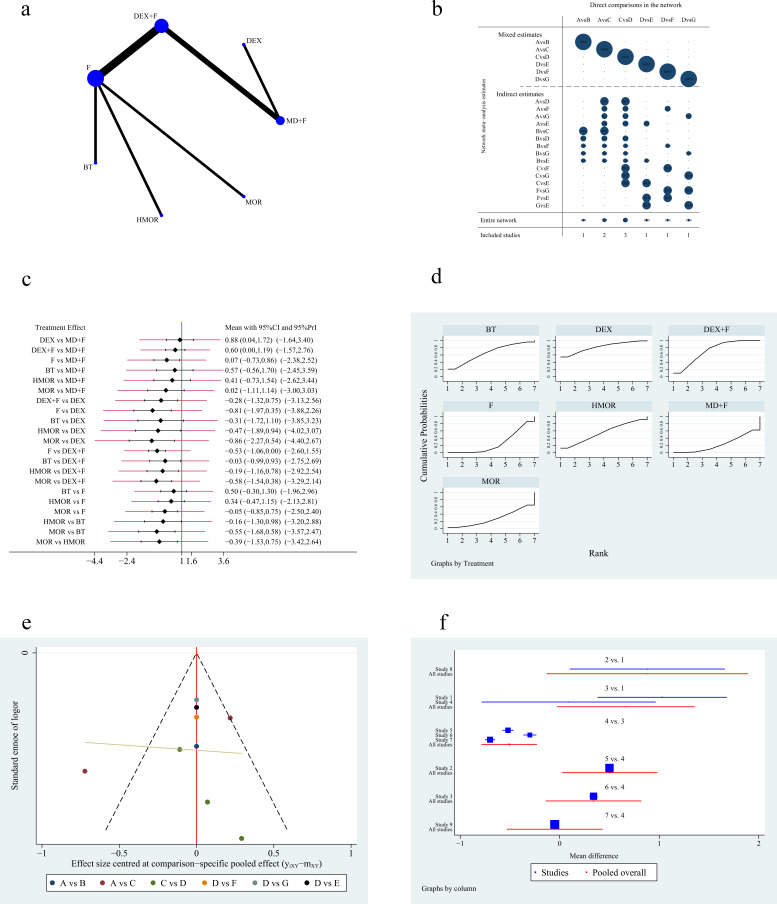
**Figures of network meta-analysis of Ramsay sedation scores**. (a) Network plot of Ramsay sedation scores. Line thickness represents the number of comparisons between the two arms, while node size represents the sample size of each arm. (b) Contribution plot of Ramsay sedation scores. (c) Prediction intervals map of Ramsay sedation scores. (d) SUCRA plot of Ramsay sedation scores. (e) Funnel plot. (f) Forest plot of Ramsay sedation scores. DEX, dexmedetomidine; F, fentanyl or its derivatives; BT, butorphanol; HMOR, hydromorphone; MOR, morphine; MD, midazolam; CI, confidence interval; PrI, prediction interval; SUCRA, the surface under the cumulative ranking curve. A: midazolam plus fentanyl or its derivatives, B: dexmedetomidine, C: dexmedetomidine plus fentanyl or its derivatives, D: fentanyl or its derivatives, E: butorphanol, F: hydromorphone, G: morphine.

RSS and VAS scores were primary efficacy outcome indexes. Out of the 14 included studies, 9 studies [3, 5, 9, 10, 11, 12, 13, 14, 15] reported RSS and involved 7 regimens. Fig. 3a shows the network plot. Fig. 3b shows the contribution plot for RSS. Paired comparisons among the seven medication regimens revealed that patients receiving dexmedetomidine during RFA for AF had higher RSS than those receiving midazolam plus fentanyl or its derivatives (mean difference [MD] –0.88, 95% CI [–0.04 to –0.72], *p* < 0.05). Dexmedetomidine plus fentanyl or its derivatives had a better sedation effect compared with fentanyl or its derivatives (MD –0.53, 95% CI [–1.06 to 0.00]), although the difference was not statistically significant (*p* > 0.05). Hydromorphone produced better sedative effects than fentanyl or its derivatives (MD 0.48, 95% CI [0.03 to 8.59]) and morphine (MD 0.48, 95% CI [0.01 to 18.54]), although, again, the difference was not statistically significant (*p* > 0.05). See Table 2 for details. The prediction intervals in the network meta-analysis (NMA) are shown in Fig. 3c. Fig. 3d shows the accumulated possibility plot using the area under the curve to indicate the likelihood of ranking first for RSS. The SUCRA plot shows that dexmedetomidine (SUCRA 81.9%) has the largest area under the curve and that the sedation effect is most likely to be better for RFA than by the other drugs, yet it was followed by dexmedetomidine plus fentanyl or its derivatives (SUCRA 71.7%), and butorphanol (SUCRA 66.2%). Midazolam plus fentanyl or its derivatives (SUCRA 23.2%) was associated with the lowest probability of high RSS. Fig. 3e presents a funnel plot to illustrate the publication bias. The overall publication bias showed a symmetrical distribution around the funnel plot, indicating low publication bias. The forest plot of RSS is shown in Fig. 3f.

**Table 2.** **NMA result of Ramsay sedation scores**.

MOR	–0.02 (–1.14, 1.11)	0.39 (–0.75, 1.53)	0.05 (–0.75, 0.85)	0.58 (–0.38, 1.54)	0.86 (–0.54, 2.27)	0.55 (–0.58, 1.68)
0.02 (–1.11, 1.14)	MD+F	0.41 (–0.73, 1.54)	0.07 (–0.73, 0.86)	0.60 (–0.00, 1.19)	0.88 (0.04, 1.72)	0.57 (–0.56, 1.70)
–0.39 (–1.53, 0.75)	–0.41 (–1.54, 0.73)	HMOR	–0.34 (–1.15, 0.47)	0.19 (–0.78, 1.16)	0.47 (–0.94, 1.89)	0.16 (–0.98, 1.30)
–0.05 (–0.85, 0.75)	–0.07 (–0.86, 0.73)	0.34 (–0.47, 1.15)	F	0.53 (–0.00, 1.06)	0.81 (–0.35, 1.97)	0.50 (–0.30, 1.30)
–0.58 (–1.54, 0.38)	–0.60 (–1.19, 0.00)	–0.19 (–1.16, 0.78)	–0.53 (–1.06, 0.00)	DEX+F	0.28 (–0.75, 1.32)	–0.03 (–0.99, 0.93)
–0.86 (–2.27, 0.54)	–0.88 (–1.72, –0.04)	–0.47 (–1.89, 0.94)	–0.81 (–1.97, 0.35)	–0.28 (–1.32, 0.75)	DEX	–0.31 (–1.72, 1.10)
–0.55 (–1.68, 0.58)	–0.57 (–1.70, 0.56)	–0.16 (–1.30, 0.98)	–0.50 (–1.30, 0.30)	0.03 (–0.93, 0.99)	0.31 (–1.10, 1.72)	BT

NMA, network meta-analysis; DEX, dexmedetomidine; F, fentanyl or its derivatives; BT, butorphanol; HMOR, hydromorphone; MOR, morphine; MD, midazolam.

**Fig. 4.**
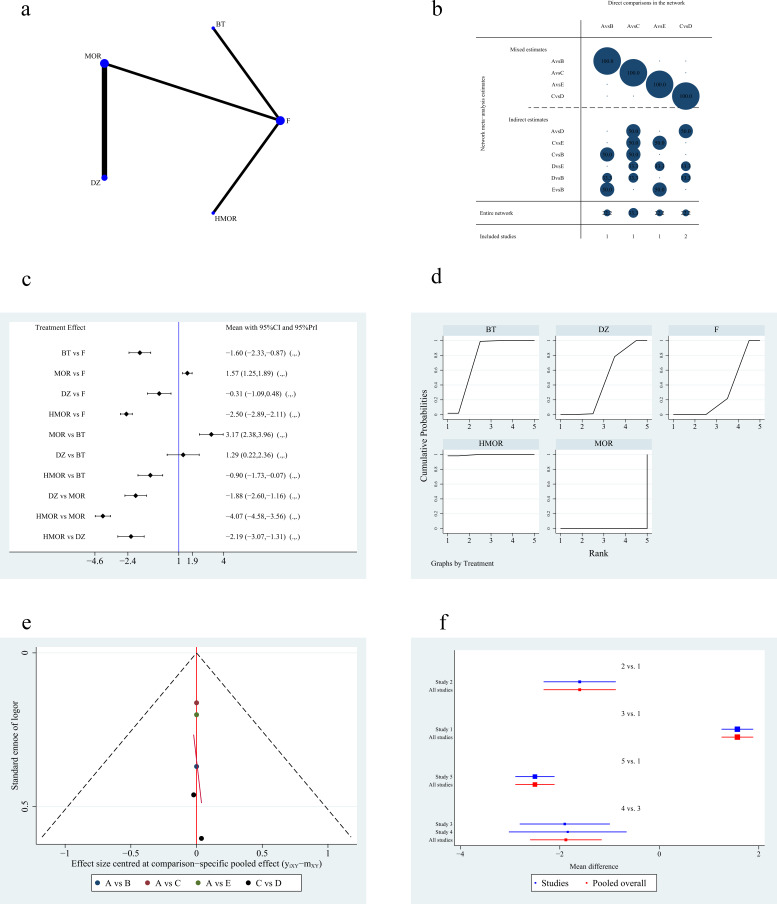
**Figures of network meta-analysis of VAS**. (a) Network plot of VAS. Line thickness represents the number of comparisons between the two arms, while node size represents the sample size of each arm. (b) Contribution plot of VAS. (c) Prediction intervals map of VAS. (d) SUCRA plot of VAS. (e) Funnel plot. (f) forest plot of VAS. F, fentanyl or its derivatives; BT, butorphanol; HMOR, hydromorphone; MOR, morphine; DZ, dezocine; CI, confidence interval; VAS, visual analog scale; SUCRA, the surface under the cumulative ranking curve; PrI, prediction interval. A: fentanyl or its derivatives, B: butorphanol, C: morphine, D: dezocine, E: hydromorphone.

Five studies [5, 6, 7, 9, 15] reported VAS scores involving five regimens. Fig. 4a,b shows the network plot and contribution plot of the analgesic scores. The NMA results showed that compared to morphine, dezocine (MD 1.88, 95% CI [1.16 to 2.60], *p* < 0.05), hydromorphone (MD 4.07, 95% CI [3.56 to 4.58], *p* < 0.05), and butorphanol (MD 3.18, 95% CI [2.38 to 3.96], *p <*0.05), fentanyl or its derivatives (MD 1.57, 95% CI [1.25 to 1.89], *p* < 0.05) had a better analgesic effect during RFA. Butorphanol (MD 2.50, 95% CI [2.11 to 2.89], *p* < 0.05) and hydromorphone (MD 1.60, 95% CI [0.87 to 2.33], *p* < 0.05) have better analgesic effects than fentanyl or its derivatives. While both dezocine (MD –2.19, 95% CI [–3.07 to –1.31], *p* < 0.05) and butorphanol (MD –0.90, 95% CI [–1.73 to –0.07], *p* < 0.05) had higher VAS scores than hydromorphone. In contrast, our results also showed that the butorphanol analgesic effect is superior to dezocine (MD –1.29, 95% CI [–2.36 to –0.22], *p* < 0.05). The analgesic effect of dezocine may be better than fentanyl or its derivatives (MD 0.31, 95% CI [–0.48 to 1.09]), however, the difference was not statistically significant (*p* > 0.05). Table 3 shows the results of the detailed analysis. The prediction intervals for NMA are shown in Fig. 4c. The SUCRA plot showed that hydromorphone (SUCRA 99.6%) had a larger area under the curve, and its analgesic effect was most likely to be superior to the other four regimens, followed by butorphanol (SUCRA 75.2%). The analgesic effect of morphine (SUCRA 0%) was most likely to rank last. See Fig. 4d for details. We mapped a funnel plot to illustrate publication bias (Fig. 4e). The scatter points in the study were relatively dispersed and had distribution associated with bias, thereby indicating that there may be some publication bias in the results. The forest plot of the VAS scores is shown in Fig. 4f.

**Table 3.** **NMA results of visual analog scale**.

MOR	–4.07 (–4.58, –3.56)	–1.57 (–1.89, –1.25)	–1.88 (–2.60, –1.16)	–3.17 (–3.96, –2.38)
4.07 (3.56, 4.58)	HMOR	2.50 (2.11, 2.89)	2.19 (1.31, 3.07)	0.90 (0.07, 1.73)
1.57 (1.25, 1.89)	–2.50 (–2.89, –2.11)	F	–0.31 (–1.09, 0.48)	–1.60 (–2.33, –0.87)
1.88 (1.16, 2.60)	–2.19 (–3.07, –1.31)	0.31 (–0.48, 1.09)	DZ	–1.29 (–2.36, –0.22)
3.17 (2.38, 3.96)	–0.90 (–1.73, –0.07)	1.60 (0.87, 2.33)	1.29 (0.22, 2.36)	BT

NMA, network meta-analysis; F, fentanyl or its derivatives; BT, butorphanol; HMOR, hydromorphone; MOR, morphine; DZ, dezocine.

### 3.3 Safety Endpoints

**Fig. 5.**
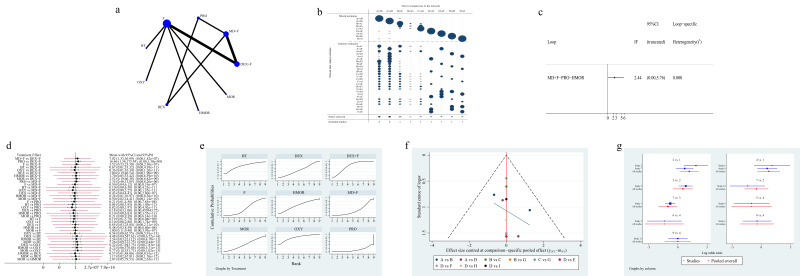
**Figures of network meta-analysis of respiratory despression**. (a) Network plot of respiratory depression. Line thickness represents the number of comparisons between the two arms, while node size represents the sample size of each arm. (b) Contribution plot of respiratory depression. (c) Inconsistency test plot of respiratory depression. (d) Prediction intervals map of respiratory depression. (e) SUCRA plot of respiratory depression. (f) Funnel plot. (g) Forest plot of respiratory depression. F, fentanyl or its derivatives; BT, butorphanol; HMOR, hydromorphone; MOR, morphine; OXY, oxycodone; PRO, propofol; DEX, dexmedetomidine; MD, midazolam; SUCRA, the surface under the cumulative ranking curve; CI, confidence interval; PrI, prediction interval. A: dexmedetomidine plus fentanyl or its derivatives, B: midazolam plus fentanyl or its derivatives, C: propofol, D: fentanyl or its derivatives, E: butorphanol, F: oxycodone, G: dexmedetomidine, H: hydromorphone, I: morphine.

The safety outcomes reported were complication rates, mainly consisting of respiratory depression, hypotension, nausea, and vomiting. For the safety outcome, 11 studies [2, 3, 4, 5, 8, 9, 10, 11, 12, 14, 15] reported rates of respiratory depression. Fig. 5a,b shows the network plot and contribution plot of the incidence of respiratory depression. We conducted inconsistencies tests based on the loop, and the results showed that the 95% CI of IF contained 0, meaning that there was no obvious inconsistency; therefore, we used the consistency model for NMA (Fig. 5c). Dexmedetomidine combined with fentanyl or its derivatives significantly reduced the incidence of respiratory depression compared with propofol (odds ratio [OR] 16.46; 95% CI [1.54 to 175.95], *p* < 0.05) and midazolam plus fentanyl or its derivatives (OR 7.02; 95% CI [1.33 to 36.99], *p* < 0.05). In our study, the rate of respiratory depression was lower in the oxycodone group than in the fentanyl or its derivatives group (OR 0.10; 95% CI [0.00 to 2.80]) and the morphine group (OR 0.10; 95% CI [0.00 to 5.57]), although the differences were not statistically significant (*p* > 0.05). See Table 4 for details. The prediction intervals for NMA are shown in Fig. 5d. The SUCRA sequencing map (Fig. 5e) indicated that by reducing the risk of respiratory depression in RFA for AF, oxycodone had the largest area under the curve and was most likely to rank as the best (SUCRA 81.4%). Butorphanol and dexmedetomidine plus fentanyl had the same SUCRA (72.2%) and were tied as being the second best. Propofol was probably the worst in terms of reducing the incidence of respiratory depression (SUCRA 10.8%). A funnel plot was drawn to illustrate the observed publication bias. Overall, publication bias showed symmetrical distribution around the funnel plot, thereby indicating low publication bias (Fig. 5f). In the funnel plot, the distribution of all the scatter points was roughly symmetric, although some research scatter points were close to the bottom of the funnel plot, thereby indicating that the results were potentially affected by publication bias and small sample effect. The forest plot is shown in Fig. 5g.

**Table 4.** **NMA results of incidence for respiratory depression**.

PRO	0.02 (0.00, 1.97)	0.21 (0.00, 9.39)	0.43 (0.07, 2.52)	0.10 (0.00, 6.83)	0.21 (0.01, 4.50)	0.06 (0.01, 0.65)	0.18 (0.03, 1.00)	0.04 (0.00, 3.95)
45.73 (0.51, 4109.80)	OXY	9.77 (0.18, 531.71)	19.49 (0.31, 1233.80)	4.72 (0.06, 378.86)	9.77 (0.36, 267.37)	2.78 (0.06, 126.04)	8.32 (0.07, 943.75)	1.86 (0.02, 216.01)
4.68 (0.11, 205.56)	0.10 (0.00, 5.57)	MOR	1.99 (0.07, 57.31)	0.48 (0.01, 18.54)	1.00 (0.11, 9.40)	0.28 (0.02, 5.36)	0.85 (0.01, 49.19)	0.19 (0.00, 11.30)
2.35 (0.40, 13.85)	0.05 (0.00, 3.25)	0.50 (0.02, 14.41)	MD+F	0.24 (0.01, 10.97)	0.50 (0.04, 6.12)	0.14 (0.03, 0.75)	0.43 (0.04, 4.41)	0.10 (0.00, 6.58)
9.69 (0.15, 641.08)	0.21(0.00, 17.02)	2.07 (0.05, 79.53)	4.13 (0.09, 187.13)	HMOR	2.07 (0.12, 36.84)	0.59 (0.02, 18.51)	1.76 (0.02, 149.60)	0.39 (0.00, 34.30)
4.68 (0.22, 98.54)	0.10 (0.00, 2.80)	1.00 (0.11, 9.40)	1.99 (0.16, 24.32)	0.48 (0.03, 8.59)	F	0.28 (0.04, 1.90)	0.85 (0.03, 25.04)	0.19 (0.01, 5.79)
16.46 (1.54, 175.95)	0.36 (0.01, 16.34)	3.52 (0.19, 66.38)	7.02 (1.33, 36.99)	1.70 (0.05, 53.42)	3.52 (0.53, 23.50)	DEX+F	3.00 (0.19, 46.54)	0.67 (0.01, 33.32)
5.49 (1.00, 30.26)	0.12 (0.00, 13.62)	1.17 (0.02, 67.81)	2.34 (0.23, 24.19)	0.57 (0.01, 48.06)	1.17 (0.04, 34.51)	0.33 (0.02, 5.18)	DEX	0.22 (0.00, 27.29)
24.61 (0.25, 2393.88)	0.54 (0.00, 62.55)	5.26 (0.09, 312.75)	10.49 (0.15, 723.40)	2.54 (0.03, 221.12)	5.26 (0.17, 160.11)	1.49 (0.03, 74.44)	4.48 (0.04, 547.62)	BT

NMA, network meta-analysis; F, fentanyl or its derivatives; BT, butorphanol; HMOR, hydromorphone; MOR, morphine; OXY, oxycodone; PRO, propofol; DEX, dexmedetomidine; MD, midazolam.

**Fig. 6.**
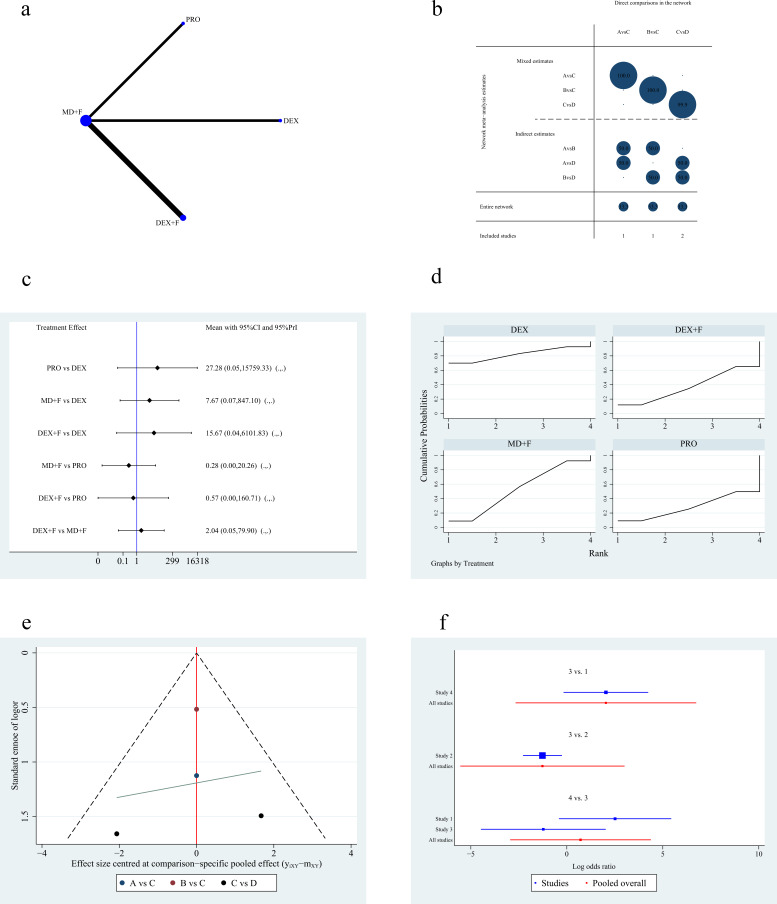
**Figures of network meta-analysis of incidence of hypotension**. (a) Network plot of incidence of hypotension. Line thickness represents the number of comparisons between the two arms, while node size represents the sample size of each arm. (b) Contribution plot of incidence of hypotension. (c) Prediction intervals map of incidence of hypotension. (d) SUCRA plot of incidence of hypotension. (e) Funnel plot. (f) Forest plot of incidence of hypotension. PRO, propofol; F, fentanyl or its derivatives; MD, midazolam; DEX, dexmedetomidine; SUCRA, the surface under the cumulative ranking curve; CI, confidence interval; PrI, prediction interval. A: dexmedetomidine, B: propofol, C: midazolam plus fentanyl or its derivatives, D: dexmedetomidine plus fentanyl or its derivatives.

Four studies [3, 4, 10, 14] reported the incidence of hypotension in RFA (Fig. 6a,b). Since there was no loop structure in the network plot, no inconsistency check was required. The NMA results (Table 5) illustrated that compared with dexmedetomidine, propofol (OR 27.27; 95% CI [0.05 to 15,721.14]), midazolam plus fentanyl or its derivatives (OR 7.66; 95% CI [0.07 to 845.55]), dexmedetomidine plus fentanyl or its derivatives (OR 15.67; 95% CI [0.04 to 6089.58]) probably had a higher occurrence of hypotension, although none of these results were statistically different (*p* > 0.05). The midazolam plus fentanyl or its derivatives group had potentially less hypotension than the dexmedetomidine plus fentanyl or its derivatives group (OR 0.49; 95% CI [0.01 to 19.11], *p >*0.05). Moreover, compared with propofol, midazolam plus fentanyl or its derivatives (OR 0.28; 95% CI [0.00 to 20.24], *p* > 0.05) and dexmedetomidine plus fentanyl or its derivatives (OR 0.57; 95% CI [0.00 to 160.50], *p* > 0.05) had a lower tendency of developing hypotension. The prediction intervals of the NMA are shown in Fig. 6c. DEX (SUCRA 88.2%) was associated with the lowest incidence of hypotension, according to the SUCRA plot, while midazolam plus fentanyl or its derivatives was the next best (SUCRA 51.9%). The propofol (SUCRA 28.8%) group had the highest incidence of hypotension according to the results of the SUCRA map (Fig. 6d). Fig. 6e shows the funnel plot. The distribution of all the scattered points in the funnel graph was symmetrical, with a small publication bias. The forest plot is shown in Fig. 6f.

**Table 5.** **NMA result of incidence of hypotension**.

PRO	0.28 (0.00, 20.24)	0.57 (0.00, 160.50)	0.04 (0.00, 21.15)
3.56 (0.05, 256.21)	MD+F	2.04 (0.05, 79.85)	0.13 (0.00, 14.40)
1.74 (0.01, 486.14)	0.49 (0.01, 19.11)	DEX+F	0.06 (0.00, 24.81)
27.27 (0.05, 15721.14)	7.66 (0.07, 845.55)	15.67 (0.04, 6089.58)	DEX

NMA, network meta-analysis; PRO, propofol; F, fentanyl or its derivatives; MD, midazolam; DEX, dexmedetomidine.

**Fig. 7.**
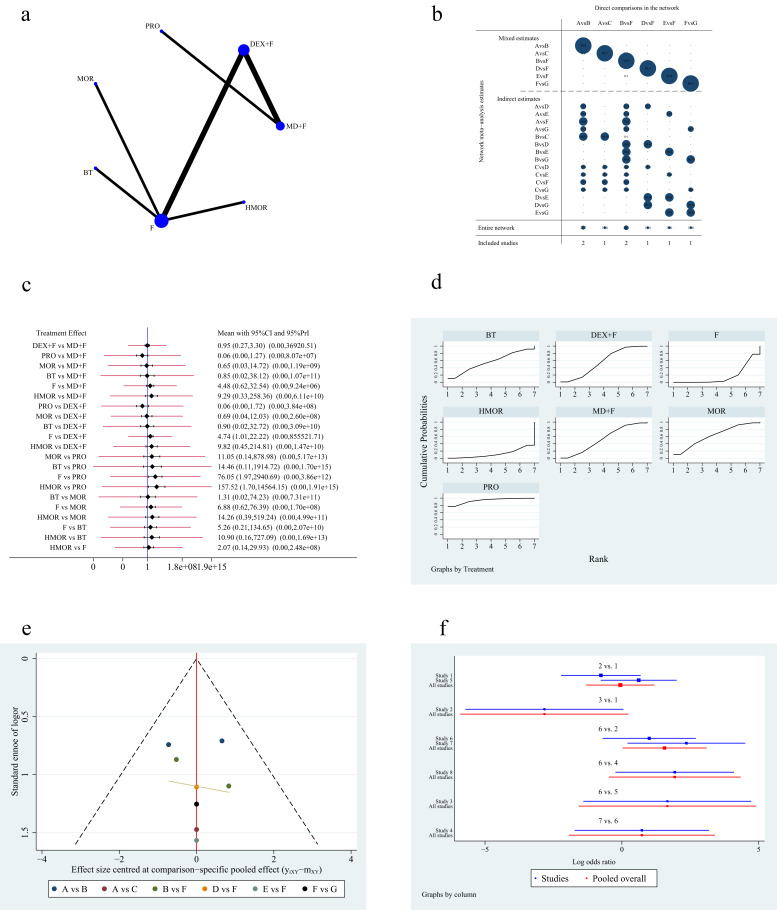
**Figures of network meta-analysis of incidence of nausea and vomiting**. (a) Network plot of incidence of nausea and vomiting. Line thickness represents the number of comparisons between the two arms, while node size represents the sample size of each arm. (b) Contribution plot of incidence of nausea and vomiting. (c) Prediction intervals map of incidence of nausea and vomiting. (d) SUCRA plot of incidence of nausea and vomiting. (e) Funnel plot. (f) Forest plot of incidence of nausea and vomiting. MD, midazolam; F, fentanyl or its derivatives; DEX, dexmedetomidine; PRO, propofol; MOR, morphine; BT, butorphanol; HMOR, hydromorphone; CI, confidence interval; PrI, prediction interval; SUCRA, the surface under the cumulative ranking curve. A: midazolam plus fentanyl or its derivatives, B: dexmedetomidine plus fentanyl or its derivatives, C: propofol, D: morphine, E: butorphanol, F: fentanyl or its derivatives, G: hydromorphone.

Eight articles [3, 4, 5, 9, 10, 12, 13, 15] reported the incidence of nausea and vomiting from seven strategies. Fig. 7a,b shows the network plot and contribution plot of the incidence of nausea and vomiting. Since there was no loop structure in the network plot, no inconsistency check was required. The NMA results showed that dexmedetomidine plus fentanyl or its derivatives reduced the incidence of nausea and vomiting compared with fentanyl alone (OR 4.74; 95% CI [1.01 to 22.22], *p* < 0.05). In addition, propofol was associated with a lower incidence of nausea and vomiting than hydromorphone (OR 0.01; 95% CI [0.00 to 0.59], *p* < 0.05) and fentanyl or its derivatives (OR 0.01; 95% CI [0.00 to 0.51], *p* < 0.05). The pairwise comparison of the remaining therapeutic regimens showed no statistical difference (Table 6). The prediction intervals for NMA are shown in Fig. 7c. The frequency analysis results from the SUCRA plots indicated that propofol (SUCRA 93.5%) reduced the incidence of nausea and vomiting. Hydromorphone was most likely to cause nausea and vomiting (SUCRA 11.5%) (Fig. 7d). The funnel plot used to assess publication bias is shown in Fig. 7e. The distribution of all scattered points in the funnel map was approximately symmetrical, although some of the research scattered points were located at the bottom of the funnel map, thereby indicating that the results may be affected by publication bias and small sample effects. The RSS forest plot is shown in Fig. 7f.

**Table 6.** **NMA results for nausea and vomiting incidences**.

PRO	11.05 (0.14, 878.91)	16.96 (0.79, 365.73)	157.52 (1.70, 14563.17)	76.05 (1.97, 2940.51)	16.04 (0.58, 441.39)	14.46 (0.11, 1914.52)
0.09 (0.00, 7.20)	MOR	1.54 (0.07, 34.69)	14.26 (0.39, 519.21)	6.88 (0.62, 76.39)	1.45 (0.08, 25.33)	1.31 (0.02, 74.22)
0.06 (0.00, 1.27)	0.65 (0.03, 14.72)	MD+F	9.29 (0.33, 258.34)	4.48 (0.62, 32.54)	0.95 (0.27, 3.30)	0.85 (0.02, 38.11)
0.01 (0.00, 0.59)	0.07 (0.00, 2.55)	0.11 (0.00, 3.00)	HMOR	0.48 (0.03, 6.97)	0.10 (0.00, 2.23)	0.09 (0.00, 6.12)
0.01 (0.00, 0.51)	0.15 (0.01, 1.61)	0.22 (0.03, 1.62)	2.07 (0.14, 29.93)	F	0.21 (0.05, 0.99)	0.19 (0.01, 4.87)
0.06 (0.00, 1.72)	0.69 (0.04, 12.03)	1.06 (0.30, 3.69)	9.82 (0.45, 214.80)	4.74 (1.01, 22.22)	DEX+F	0.90 (0.02, 32.72)
0.07 (0.00, 9.16)	0.76 (0.01, 43.35)	1.17 (0.03, 52.46)	10.90 (0.16, 727.04)	5.26 (0.21, 134.64)	1.11 (0.03, 40.25)	BT

NMA, network meta-analysis; MD, midazolam; F, fentanyl or its derivatives; DEX, dexmedetomidine; PRO, propofol; MOR, morphine; BT, butorphanol; HMOR, hydromorphone.

## 4. Discussion

RFA is an effective treatment strategy for AF, and its success is related to its analgesic and sedative effects. However, analgesic and sedative drugs often lead to adverse reactions. Therefore, selecting an effective and safe drug regimen is crucial. Thus, we conducted this network meta-analysis to compare the efficacy and safety of various analgesic and sedative strategies during RFA for AF. We identified 14 studies, included in NMA; however, all the outcome indicators were only reported in a few studies. Our analyses confirmed several findings: For the analgesic effect, hydromorphone and butorphanol were more prominent, and both dezocine and fentanyl or its derivatives provided better analgesia effects than morphine. For the sedative effect, dexmedetomidine had a higher sedation score compared to midazolam plus fentanyl or its derivatives. In terms of safety endpoints, compared with dexmedetomidine plus fentanyl or its derivatives, propofol and midazolam plus fentanyl or its derivatives were associated with an increased incidence of respiratory depression, while propofol had a lower incidence of nausea and vomiting than hydromorphone and fentanyl or its derivatives. Dexmedetomidine plus fentanyl or its derivatives leads to a lower incidence of nausea and vomiting than fentanyl or its derivatives. There was no statistically significant difference in the incidence of hypotension among these various regimens during RFA for AF.

Opioids, which mainly act on opiate µ, κ, and δ receptors, have been widely used in RFA for AF. Hydromorphone, a semisynthetic opioid analgesic, has been widely used for intraoperative and postoperative analgesia and cancer analgesia [16, 17, 18]. It mainly acts on opioid µ receptors in the central nervous system. Its chemical structure is based on morphine, thereby oxidizing the 6-position hydroxyl groups to ketone carbonyl groups and reducing the 7-position and 8-position double bonds. Such a structure increases its lipoid solubility and analgesic efficacy. The ranking results showed that the analgesic effect of butorphanol was second only to hydromorphone. The analgesic effect of butorphanol had been confirmed as being about five times that of morphine, while the incidence of respiratory depression was one-fifth that of morphine, which is consistent with the trend in our results. Butorphanol, when used as a novel hybrid opioid receptor agonist or antagonist, can produce analgesia and sedation by activating κ_1_ receptors in the spinal cord, and partially blocking the µ receptors. Activation of the µ opioid receptors can reduce the sensitivity of chemoreceptors to ${\mathrm{CO}}_{2}$ and inhibit respiratory function in a dose-dependent manner. This may be the reason why butorphanol, compared with other opioids, has better sedative and analgesic effects, while not increasing the incidence of respiratory depression [5]. The ranking graph showed that the tendency for oxycodone to develop respiratory depression was lower than for butorphanol. Studies have shown that the incidence of intraoperative respiratory depression and hypoxemia of oxycodone was significantly lower than for fentanyl [19]. Oxycodone could activate µ and κ receptors. Activation of the κ receptor can inhibit respiratory depression that has been mediated by the µ receptor [20], which explains why oxycodone has less respiratory depression than fentanyl or its derivatives. Hydromorphone leads to the lowest incidence of nausea and vomiting (*p* > 0.05). However, more high-quality RCTs are needed to verify these results. In addition, part of opioid metabolism may be influenced by genetics and ethnicity. Chinese individuals have higher morphine metabolic rates, while no significant differences were found in the metabolism of oxycodone, hydromorphone, and fentanyl [21]. The metabolism of oxycodone may be related to polymorphic genetic enzymes CYP2D6 and CYP3A. Race has no significant effect on the pharmacokinetics of oxycodone [22]. However, it is necessary to perform multi-racial, multi-center, and large-sample clinical trials to verify these findings.

Dexmedetomidine is a novel α_2_ receptor agonist, which is used in RFA for AF. Our efficacy ranking results suggest that the sedative effect of dexmedetomidine plus fentanyl or its derivatives may be better than that of fentanyl alone and worse than by dexmedetomidine. The combination of dexmedetomidine and fentanyl had lower rates of respiratory depression, nausea, and vomiting than either drug administered alone. Dexmedetomidine acts on α2 adrenergic receptors in the locus coeruleus of the central nervous system and the spinal cord. The locus coeruleus is an important center for the maintenance of arousal and produces sedation without affecting the respiratory center. Meta-analyses and randomized controlled clinical trials have shown that dexmedetomidine and opioids have synergistic analgesic effects, which can reduce the dosage of opioids [23, 24, 25]. Due to dose dependence in the occurrence of adverse reactions, dexmedetomidine combined with opioids can reduce the incidence of nausea, vomiting, respiratory depression, and other adverse reactions compared with opioids alone. Studies have shown that the incidence of intraoperative hypotension is significantly increased in the dexmedetomidine group compared with the non-dexmedetomidine group during cardiac surgery and non-cardiac surgery [25]. In addition, dexmedetomidine can stabilize perioperative hemodynamics, and when combined with fentanyl, dexmedetomidine can inhibit the reduction in blood pressure during anesthesia induction [26, 27, 28]. Our NMA of the incidence of hypotension during RFA for AF showed no significant statistical difference, which may be due to the small number of included studies and publication bias. Moreover, studies have shown that there are differences in the metabolism of dexmedetomidine between White and Black races. The plasma dexmedetomidine concentrations in Black people are higher than in White people [29]. However, Black and White individuals have similar sympathetic and cardiovascular responses to dexmedetomidine. Nevertheless, there are interindividual differences in the responses to dexmedetomidine that remain unexplained. A study of dexmedetomidine intolerance/failure in mechanically ventilated adults showed that non-black races were an independent predictor of intolerance/failure. These results suggest that racial differences affect dexmedetomidine reactivity and metabolism [30]. However, most of the subjects included in the network meta-analysis were Asian, which may reduce the interpretability of the results.

Midazolam is the main benzodiazepine used in RFA for AF. Midazolam works primarily by increasing the frequency of the chloride channel openings to enhance the action of the inhibitory neurotransmitter γ-aminobutyric acid (GABA) receptor in the central nervous system. It has sedative and hypnotic effects without analgesic effects, although when used in combination with opioids, it can enhance the analgesic effects of opioids. Therefore, midazolam is more commonly used in combination with other opioids or anesthetics. Clinical RCTs have shown that midazolam combined with opioids can provide good sedative and analgesic effects, and relieve dyspnea in cancer patients [31, 32]. Sedation using benzodiazepines in combination with opioids can increase the incidence of respiratory depression. However, the ranking results of our NMA showed that midazolam plus fentanyl or its derivatives had a higher incidence of respiratory depression than dexmedetomidine plus fentanyl or its derivatives (*p <* 0.05). Compared with midazolam plus fentanyl or its derivatives, fentanyl or its derivatives had a higher ranking in terms of sedative efficacy and a lower incidence of respiratory depression. This result was produced by indirect comparison and had no statistical significance (*p* > 0.05), thus, it should be interpreted with caution. No head-to-head randomized controlled trials comparing sedation and safety of midazolam combined with opioids versus opioids in radiofrequency ablation for atrial fibrillation were found. Therefore, more high-quality RCTs are needed to confirm this finding. In addition, midazolam can reduce vascular resistance and arterial pressure, while increasing the heart rate [33]. The metabolic differences in midazolam across ethnic groups remain controversial. A previous study has shown that there were no statistical differences in midazolam metabolism between Japanese and European populations [34]. However, a study on midazolam among five ethnic populations in China has shown significant differences in midazolam metabolism rates [35].

Propofol, an anesthetic drug that does not have analgesic effects, is used for deep sedation during RFA for AF. Propofol combined with midazolam provides good efficacy and safety in electrical cardioversion [36]. However, propofol presents a risk of dose-related respiratory depression and excessive sedation. NMA showed that propofol deep sedation had lower rates of respiratory depression and hypotension in RFA, yet higher rates of nausea and vomiting. There were only two studies related to propofol, thus, the effectiveness and safety of propofol in RFA for AF need to be verified.

Our study has a few potential limitations. First, we conducted integrated analyses of fentanyl and its derivatives, ignoring the differences and connections between fentanyl, sufentanil, alfentanil, and remifentanil, which may have weakened the credibility of the results. Second, most studies did not report using the blind method and allocation hiding in detail, and some studies did not clarify whether the allocation was random. Third, there were fewer than three studies that included propofol, hydromorphone, oxycodone, and dezocine, and the funnel plot suggested that publication bias might have affected the analysis of these results. Fourth, there was no statistical difference between most indirect comparisons, meaning that the results should be interpreted with caution. Therefore, there is still a need for more high-quality randomized controlled clinical trials to validate these results. Finally, the research populations included in this network meta-analysis were mostly Asian. There are differences in drug response and metabolism among the different ethnic populations, which could affect the interpretation of the network meta-analysis results. Notwithstanding these limitations, the findings from this NMA represent the most current comprehensive available database to guide the use of analgesic and sedative drugs during RFA for AF.

## 5. Conclusions

We compared the efficacy and safety of ten analgesic or sedative regimens during RFA for AF. In terms of efficacy, hydromorphone, butorphanol, and dezocine had better analgesic effects than fentanyl. Dexmedetomidine had better sedative effects. In terms of safety, dexmedetomidine, oxymorphone, and butorphanol had the best scores. It is necessary to explore the regimen that can consider both effectiveness and safety during radiofrequency ablation for atrial fibrillation.

## Data Availability

The datasets used and/or analyzed during the current study are available from the corresponding author on reasonable request.

## Supplementary Material

Supplementary material associated with this article can be found, in the online version, at https://doi.org/10.31083/j.rcm2501012.
